# Haplotype of the *Interleukin 17A* gene is associated with osteitis after Bacillus Calmette-Guerin vaccination

**DOI:** 10.1038/s41598-017-12113-z

**Published:** 2017-09-15

**Authors:** Matti Korppi, Johanna Teräsjärvi, Milla Liehu-Martiskainen, Eero Lauhkonen, Juho Vuononvirta, Kirsi Nuolivirta, Liisa Kröger, Laura Pöyhönen, Minna K. Karjalainen, Qiushui He

**Affiliations:** 1 Center for Child Health Research, University of Tampere and University Hospital, Tampere, Finland; 2 Department of Medical Microbiology and Immunology, University of Turku, Turku, Finland; 3 Department of Pediatrics, Seinäjoki Central Hospital, Seinäjoki, Finland; 4 Department of Pediatrics, Kuopio University Hospital, Kuopio, Finland; 5 St. Giles Laboratory of Human Genetics of Infectious Diseases, Rockefeller Branch, The Rockefeller University, New York, NY USA; 6 PEDEGO Research Unit and Medical Research Center Oulu, University of Oulu, Oulu, Finland; 7 Department of Children and Adolescents, Oulu University Hospital, Oulu, Finland; 8 Department of Medical Microbiology, Capital Medical University, Beijing, China

## Abstract

Bacillus Calmette-Guerin (BCG) osteitis was more common in Finland than elsewhere at the time when universal BCG vaccinations were given to Finnish newborns. There is evidence that IL-17 plays a role in the defense against tuberculosis. The aim of this study was to evaluate the associations of *IL17A* rs4711998, *IL17A* rs8193036 and *IL17A* rs2275913 single-nucleotide polymorphisms (SNPs) with the risk of BCG osteitis after newborn vaccination. *IL17A* rs4711998, rs8193036 and rs2275913 SNPs were determined in 131 adults had presented with BCG osteitis after newborn BCG vaccination. We analyzed, using the HaploView and PLINK programs, whether allele or haplotype frequencies of these SNPs differ between the former BCG osteitis patients and Finnish population controls. Of the three *IL17A* SNPs studied, rs4711998 associated nominally with BCG osteitis; minor allele frequency was 0.215 in 130 BCG osteitis cases and 0.298 in 99 controls (p = 0.034). Frequency of the second common haplotype (GTA) differed significantly between BCG osteitis cases and controls (0.296 *vs*. 0.184, p = 0.040 after multi-testing correction). The GTA haplotype of the *IL17A* SNPs rs4711998, rs8193036 and rs2275913 was associated with osteitis after BCG vaccination.

## Introduction

Interleukin-17A (IL-17A) belongs to pro-inflammatory cytokines produced by T helper 17 (Th17) cells^1,2^. Th17 cells and Th17-derived cytokines participate in the defense against different pathogens, like *Mycobacterium tuberculosis* ^1^. In addition, Th17 cells and Th17-derived cytokines are involved in the pathogenesis of diseases presenting with chronic inflammation, such as asthma and autoimmune diseases^2^.

Six studies were included in a systematic review on the role of IL-17A in active and latent tuberculosis (TB), and three studies on the role of IL-17A in Bacillus Calmette-Guerin (BCG) vaccination^3^. The authors concluded that IL-17A acts as an effector protective molecule in patients with TB and in children after BCG vaccination^3^.

In a recent meta-analysis, the *IL17A* rs3748067 single-nucleotide polymorphism (SNP) was associated with the susceptibility to TB in Asian populations^4^. There was no significant association between *IL17A* rs2275913 polymorphism and TB risk, but a significant association was documented in two later studies in Chinese^5^ and Brazilian^6^ populations. The meta-analysis did not include the *IL17A* rs8193036 and *IL17A* rs4711998 SNPs^4^, which we selected in addition to *IL17A* rs2275913 for the present study.

BCG osteitis was more common in Finland than elsewhere at the time when BCG vaccinations were given to all Finnish newborns. During the years 1960–1988, altogether 222 children suffered from BCG osteitis in infancy^7,8^, and in 2008–2009, we collected questionnaire data from 160 of them and blood samples from 132 of them for immunological and genetic studies^9,10^.

The aim of the present study was to evaluate the association between the *IL17A* rs4711998 (−877A > G), *IL17A* rs8193036 (−737C > T) and *IL17A* rs2275913 (−197G > A) SNPs and the risk of BCG osteitis after newborn vaccination. We compared the allele and haplotype frequencies of the three SNPs between 131 former BCG osteitis patients and 99 Finnish population controls obtained from the 1000 Genomes Project^11^.

## Results

Of the three *IL17A* SNPs, rs4711998 associated nominally with BCG osteitis (Table 1). The minor allele was protective (minor allele frequency, MAF, cases/controls 0.215/0.298, OR = 0.61, p = 0.034). However, the statistical significance was lost after multi-testing correction. We further analyzed the three-SNP haplotypes for association with BCG osteitis, and detected that the second common haplotype, GTA consisting of major alleles for rs4711998 (G) and rs8193036 (T), and the minor allele A for rs2275913, was overrepresented in BCG osteitis cases compared to controls (0.291 *vs*. 0.184, p = 0.008) (Table 2). This association remained significant after multi-testing correction.

**Table 1:** Association of IL17A single-nucleotide polymorphisms (SNPs) with BCG osteitis SNP, single-nucleotide polymorphism; MAF, minor allele frequency; OR, odds ratio, CI, confidence interval. OR for the minor allele in logistic regression under the additive model. P value 0.034 not significant after multiple-testing correction.

**SNP**	**Minor allele**	**Case/control MAF**	**OR (95% CI)**	**p value**
rs4711998	A	0.215/0.298	0.61 (0.39–0.96)	0.034
rs8193036	C	0.350/0.394	0.85 (0.59–1.21)	0.364
rs2275913	A	0.500/0.429	1.33 (0.91–1.93)	0.138

**Table 2:** Association of *IL17A* three-SNP haplotypes with BCG osteitis. Haplotypes formed by alleles of *IL17A* SNPs rs4711998, rs8193036 and rs2275913. *P* value 0.008 significant after multiple-testing correction with 100,000 permutations (*p* = 0.040).

**Haplotype**	**Haplotype frequency**	**Case/control haplotype frequency**	***p*** **value**
GTG	0.275	0.254/0.302	0.255
GTA	0.245	0.291/ 0.184	0.008
GCA	0.115	0.111/0.120	0.748
GCG	0.114	0.128/0.095	0.278
ACA	0.088	0.074/0.107	0.210
ATG	0.087	0.068/0.112	0.101
ACG	0.054	0.044/0.066	0.307
ATA	0.022	0.029/0.013	0.235

The results suggest that the minor allele A of the SNP *IL17A* rs4711998 may be protective for complications like osteitis after BCG vaccination, and that the associated GTA haplotype may be predisposing.

## Discussion

The main result of the present study was that the haplotype GTA of *IL17A* rs4711998, rs8193036 and rs2275913 polymorphisms was more common in former BCG osteitis patients than in the Finnish population controls. The difference between cases and controls remained significant after multi-testing correction. There was also a nominally significant difference between cases and controls in the allele frequency of *IL17A* rs4711998. The major allele G was the predisposing and the minor allele A the protective allele.

As published recently from this cohort, the minor allele A and the variant genotypes GA and AA of the *IL17A* rs2275913 were more common in BCG osteitis patients than in 405 healthy infants from South-West Finland^12^. The result is in agreement with our present result, though now the most significant polymorphism was *IL17A* rs4711998.

Six studies were included in a recent systematic review on the role of IL-17A in active and latent TB disease^3^, and the conclusion was that IL-17A was low in active TB but increased during the conversion from active to latent TB. Three studies included in the meta-analysis evaluated the role of IL-17A in BCG vaccination^3,13–15^, and BCG vaccination induced high serum levels of IL-17A. Thus, IL-17A seems to act as an effector protective molecule like interferon gamma (IFN-γ) in patients with TB and in children after BCG vaccination^3^.

No previous studies are available on the association of *IL17A* gene polymorphisms with responses to BCG vaccination or with complications after BCG vaccination. Instead, some data are available on different *IL17* polymorphisms and TB risk, as summarized in a recent meta-analysis^4^. *IL17A* rs3748067 and *IL17F* rs763780 SNPs were associated with the susceptibility to TB in Asian populations, but not in European populations. *IL17A* rs2275913 polymorphism, which was determined in the present study, had no such association, and the *IL17A* rs4711998 and rs8193036 SNPs, which also were determined in the present study, were not reviewed. Recently, *IL17A* rs2275913 polymorphism was associated with the risk of TB in the Chinese^5^ and Brazilian^6^ populations. *IL17A* rs8193036 polymorphism was determined in the Brazilian study, but the data did not fulfill the HWE criteria and were not analyzed^6^. *IL17A* haplotypes were not evaluated in any of the previous studies.

The strength of the present study is the unique material, 130 patients who presented with firmly diagnosed BCG osteitis after BCG vaccination as newborns. This material is the largest one ever published on BCG infections. All BCG osteitis cases were ethnic Finns, and this homogeneity is a benefit in genetic studies. Originally, the total number of BCG osteitis patients was 222, and as published recently, none of them had died of tuberculosis, BCG infection or other severe infection^10^. Thus, the present sample of 130 former BCG patients probably represents well the original BCG osteitis patients at the time when BCG vaccination was offered to all Finnish newborns. Currently, only risk groups are vaccinated in Finland.

The main shortcoming of the study is the small number of patients and controls for genetic analyses. Thus, there is a need for replication of our findings in a larger cohort and in other populations. However, a collection of a BCG osteitis cohort that is larger than the present is not realistic. Therefore, an association between the GTA haplotype of the three *IL17A* SNPs and other Th17-associated phenotypes, if present, would offer indirect evidence for our present observations. In addition, we could not include any confounding factors in the logistic regression, because the controls were from the publicly available 1000 Genomes Finnish population^11^ and no clinical data are available for these controls. As there were no genome-wide SNP data available, assessing population stratification by genetic methods was not possible. However, since both cases and controls were from the Finnish population, it is unlikely that population stratification would have any substantial impact on the results.

We included three *IL17A* polymorphisms in the study, but the functions of these SNPs are poorly known. The association between *IL17A* rs2275913 polymorphism and IL-17A production has been documented in cell cultures^16^ and in healthy infants^17^, but the results were inconclusive. The *IL17A* rs8193037 polymorphism was associated with plasma IL-17A level in adults with congestive heart failure^18^. The *IL17A* rs8193036 polymorphism influenced IL-17A production in blood cells obtained from adults with inflammatory bowel disease^19^. No data are available on the association between the *IL17A* rs4711998 polymorphism and IL-17A production.

Instead, numerous studies are available on the association of these SNPs with asthma and inflammatory diseases. In three meta-analyses, the higher asthma risk was associated with *IL17A* rs8193036^20^, rs2275916^21^ and rs4711998^22^ polymorphisms. In two meta-analyses, the *IL17A* rs2275913 SNP increased the risk of rheumatic diseases^23^, and *IL17A* polymorphisms, when analyzed as combined, were associated with the risk of ulcerative colitis^24^. Most studies were done in Asian populations, and therefore, the results cannot be directly applied to other such as European populations.

In conclusion, the GTA haplotype of *IL17A* rs4711998, *IL17A* rs8193036 and *IL17A* rs2275913 was significantly associated with osteitis after BCG vaccination as newborn.

## Material and Methods

As published previously, we collected whole blood samples from 132 Finnish former BCG osteitis patients, and sent the samples to the laboratory of the National Institute for Health and Welfare, Turku, Finland^9,10^. DNA was isolated from 200 μL of whole blood, and DNA samples were frozen at −70 °C. For the present study, the samples were transferred to the laboratory of Medical Microbiology and Immunology, University of Turku, Turku, Finland, where *IL17A* rs4711998, rs8193036 and rs2275913 SNPs were determined. The reason, why we included just these three SNPs in this study, is the available information on their association with many diseases. As summarized in meta-analyses^20–24^, *IL17A* rs2275913 and rs8193036 SNPs have been associated with infectious, allergic, rheumatic and even malignant diseases. *IL17A* rs4711998 has been often included in these studies, though such associations have been rare.

BCG osteitis in the study subjects was diagnosed by BCG culture and/or typical histology during years 1960–1988 when the study subjects were infants^7,8^. This is the largest BCG osteitis material ever published. At that time, more than 95% of all newborns received the BCG vaccination in Finland, and the diagnostics of severe complications like BCG osteitis was centralized in the Public Health Institute, Helsinki, Finland.

### Controls

Controls for the *IL17A* rs4711998, rs8193036 and rs2275913 polymorphisms were obtained from the Finnish population data (N = 99) of the 1000 Genomes Project^11^.

### Genotyping

All 132 BCG osteitis patients were genotyped for *IL17A* rs2275913 gene (−197G > A) by a newly developed high resolution melting analysis (HRMA) (Roche Diagnostics Light Cycler 480, Basel, Switzerland). The primers (forward 5′-TCTGCCCTTCCCATTTTCCTTC-3′ and reverse 5′-GGTTAAAATTTCCGCCCCCAATT-3′) were designed with Primer-Blast design tool. The known *IL17A* rs2275913 standards (wild type, heterozygote and homozygote variants) were used in each run. The method for *IL17A* rs2275913 was published in detail recently^25^.

Likewise, HRMA was used for genotyping of 130 BCG osteitis patients for *IL17A* rs4711998 (−877 A > G) and rs8193036 (−737 C > T) SNPs. HRM analyses were performed by LightCycler 480 version 5.1 (Roche, Basal, Switzerland) using SensiFAST HRMA melting master kit (Bioline, London, UK). In each run, the volume was 20 µl consisting of 4 µl genomic DNA (8.0 ng/µl) and 16 µl of master mix including 10 µl melting master mix and 0.2 µM of forward and reverse primers. Primers for the *IL17A* rs4711998 and rs8193036 SNPs were designed with Primer-Blast design tool (http://www.ncbi.nlm.nih.gov/tools/primer-blast/) and were ordered from Sigma-Aldrich Company (Saint Louis, Missouri, USA). High Performance Liquid Chromatography (HPLC) quality primers used in the HRMA analysis were as follows: *IL17A* rs4711998 forward 5′-TCTTGTCCTAGTCCTCTGTATTC-3′ and reverse 5′-GTAAGATGAACTTGGACTCAGGTC-3′, and *IL17A* rs8193036 forward 5′-CTCCTTTCTAGTTCTCATCACTCTC-3′ and reverse 5′-GGGGATAGAGACTGGACAAA-3′.

HRMA polymerase chain reaction (PCR) program started with an initial denaturation of 3 min at 95 °C followed by 39 cycles amplification of 5 s at 95 °C and annealing of 10s at 61 °C (*IL17A* rs8193036) or at 60 °C (*IL17A* rs4711998) and extension of 15 s at 72 °C. After the PCR step, high resolution melting cycle conditions were as outlined by Roche: first heated to 95 °C and held for 1 min, cooled to pre-hold temperature (40 °C) followed by melting interval for collecting fluorescence from 60 °C to 95° at ramp rate of 0.2 °C per second.

Based on the HRMA melting profiles of the SNPs two samples were selected to represent each genotype. The samples were sequenced in the Finnish Institute for Molecular Medicine (FIMM), Helsinki, Finland, to confirm the identity of the sequences. These samples were used as positive controls on each run.

### Function

The association between *IL17A* rs2275913 (−197G > A) polymorphism and IL-17A production has been documented in healthy adults^16^ and in healthy infants^17^, though the findings suggested different directions for the influence. *In vitro* stimulated T cells from the adults possessing the variant A allele produced significantly more IL-17 than those without the A allele^16^, whereas serum IL-17A concentrations were high in infants with the wild GG, intermediate in infants with the variant GA, and low or unmeasurable in infants with the variant AA genotype^17^. The presence of the variant GA or GG genotype of the *IL17A* rs8193037 (−737C > T) was associated with lower plasma IL-17A levels compared to wild genotypes in adults with congestive heart failure, but the *IL17A* rs2275913 had no such association^18^. The *IL17A* rs8193036 influenced the DNA methylation, and mRNA and protein expression of IL-17A in blood cells obtained from adults with inflammatory bowel disease^19^. No data are available on the association between the *IL17A* rs4711998 (−877A > G) SNP and IL-17A production.

### Statistical analyses

The *IL17A* rs4711998, rs8193036 and rs2275913 SNPs were analyzed for association with BCG osteitis using logistic regression under the additive model with PLINK, version 1.09^26^. Estimation of haplotype frequencies and haplotype association analysis with the χ^2^ test were performed with Haploview, version 4.3, 0^27^ and multiple-testing correction was done with 100,000 permutations. The *IL17A* rs4711998, rs8193036 and rs2275913 SNPs did not deviate from Hardy-Weinberg Equilibrium (HWE). Genotyping rates for the three SNPs were 0.992 (rs4711998), 0.992 (rs8193036), and 0.985 (rs2275913) indicating good genotyping quality, and no individuals were excluded.

### Ethics

The study was carried out in accordance with the approved guidelines of the WMA Declaration of Helsinki. The study was accepted by the Ethics Committee of the Tampere University Hospital District. Written informed consent was obtained from the study subjects, including permission to perform genetic studies concerning susceptibility to mycobacterial infections. The samples were studied in the laboratories as anonymized and coded.
